# IS GHANA READY FOR GERIATRICS? MEDICAL STUDENTS’ INTEREST AND INTENTION TOWARD GERIATRIC SPECIALIZATION IN GHANA

**DOI:** 10.1093/geroni/igz038.1097

**Published:** 2019-11-08

**Authors:** Grace Karikari, Lesa L Huber, David K Lohrmann

**Affiliations:** Indiana University School of Public Health, Bloomington, Indiana, United States

## Abstract

Aging is a global phenomenon, and the population of elderly persons continues to increase in many communities around the world. Ghana is not an exception. As the demographics are changing, it is vital that health care providers are trained to effectively diagnose, care for, and manage aging-related conditions to improve the quality of life of the elderly. The aim of this study is to explore the underlying reasons for medical students’ interest in geriatrics and their intentions, likelihood of selecting geriatrics as a specialty for future practice. This exploratory study involved (n =12) fourth, fifth and sixth year medical students in a Ghanaian public university. Semi-structured interviews guided by the theory of planned behavior were conducted. The findings show a lack of geriatric education or training for students in this institution and region. Students’ interest and preferences for geriatrics were mainly influenced by the lack of knowledge and exposure to the specialty, as well as what it entailed to be a geriatrician. Although some students expressed interest in working with older patients, none had the intention to pursue geriatrics as a career option. Creating positive exposure to geriatrics through educational and vocational opportunities are necessary. The findings from this study can be instrumental in developing a knowledge-base of aging and caring for the elderly. Findings can also serve as the foundation for a more comprehensive study, leading to effective geriatrics education interventions in Ghana and Africa at large.

